# Identification of a Novel *Brevibacillus laterosporus* Strain With Insecticidal Activity Against *Aedes albopictus* Larvae

**DOI:** 10.3389/fmicb.2021.624014

**Published:** 2021-02-17

**Authors:** Giulia Barbieri, Carolina Ferrari, Stefania Mamberti, Paolo Gabrieli, Michele Castelli, Davide Sassera, Emanuela Ursino, Viola Camilla Scoffone, Giacomo Radaelli, Emanuela Clementi, Luciano Sacchi, Eugenio Ferrari, Giuliano Gasperi, Alessandra M. Albertini

**Affiliations:** ^1^Department of Biology and Biotechnology “Lazzaro Spallanzani”, University of Pavia, Pavia, Italy; ^2^Department of Biosciences, University of Milan, Milan, Italy

**Keywords:** *Aedes albopictus*, *Brevibacillus laterosporus*, biopesticides, genome sequencing, soil microbiota community

## Abstract

Bacterial species able to produce proteins that are toxic against insects have been discovered at the beginning of the last century. However, up to date only two of them have been used as pesticides in mosquito control strategies targeting larval breeding sites: *Bacillus thuringensis* var. *israelensis* and *Lysinibacillus sphaericus*. Aiming to expand the arsenal of biopesticides, bacterial cultures from 44 soil samples were assayed for their ability to kill larvae of *Aedes albopictus*. A method to select, grow and test the larvicidal capability of spore-forming bacteria from each soil sample was developed. This allowed identifying 13 soil samples containing strains capable of killing *Ae. albopictus* larvae. Among the active isolates, one strain with high toxicity was identified as *Brevibacillus laterosporus* by 16S rRNA gene sequencing and by morphological characterization using transmission electron microscopy. The new isolate showed a larvicidal activity significantly higher than the *B. laterosporus* LMG 15441 reference strain. Its genome was phylogenomically characterized and compared to the available *Brevibacillus* genomes. Thus, the new isolate can be considered as a candidate adjuvant to biopesticides formulations that would help preventing the insurgence of resistance.

## Introduction

The vector control market, which includes chemical, biological, and physical (e.g., UV lights and traps) strategies, was valued at USD 15.12 billion in 2017 and is projected to reach 20.37 billion by 2023. The segment likely to grow at the highest rate is the one of biological control: globally the biopesticide market has an estimated value of USD 4.3 billion in 2020, and it is expected to reach USD 8.5 billion in the next five years (Markets and markets, 2018). This dramatic increase can be attributed to the growing concerns on the environmental impact of chemical insecticides and the continuous increase of insecticide-resistance populations in many vector species. Moreover, unlike conventional insecticides which often target a broad spectrum of insects, including beneficial species such as pollinators, bioinsecticides provide a more targeted activity toward selected species. Furthermore, these products are quickly biodegraded, leaving virtually no harmful residues and having limited long-term impact on the environment.

The biocontrol of mosquitoes began with the identification of bacteria active against Diptera, specifically *Bacillus thuringiensis* var. *israelensis* in 1977 (Goldberg and Margalit, 1977) and *Lysinibacillus sphaericus* strain 1593 (Singer, 1973). These two bacterial species achieved commercial success and are now broadly used.

The toxic activity of *B. thuringiensis* var. *israelensis* is due to four major crystal proteins, Cry4Aa, Cry4Ba, Cry11Aa, and Cyt1Aa, whose genes reside on a 128 kb plasmid (pBtoxis) (Berry et al., 2002; Ben-Dov, 2014). Other proteins, such as Cry10Aa, Cyt2Ba, Cyt1Ca, P19, and P20, contribute to the toxicity of *B. thuringiensis* var. *israelensis* (Palma et al., 2014). The larvicidal activity of the Cry and Cyt toxins is due to their ability to perforate midgut epithelial cells membranes, with the Crys binding to membrane receptors (Feldmann et al., 1995) and Cyt1Aa binding unsaturated phospholipids (Thomas and Ellar, 1983; Du et al., 1999). The major pitfalls of *B. thuringiensis* var. *israelensis* is its sensitivity to UV-damage (Myasnik et al., 2001), which required the development of stabilizing formulations (Lacey, 2007) and biotechnological approaches, including transgenic crops or recombinant bacteria expressing *B. thuringiensis* toxins (Federici et al., 2003; Sanahuja et al., 2011; Ursino et al., 2020).

One case of mosquito population resistant to *B. thuringiensis* var. *israelensis* has been described (Paul et al., 2005); however, most targeted studies have reported no resistance after long periods of treatment (Goldman et al., 1986; Becker and Ludwig, 1993). One of the obstacles to the development of resistance to *B. thuringiensis* var. *israelensis* is the synergism between Cry toxins and Cyt1Aa, with the latter functioning as a surrogate receptor able to promote toxin binding to host target membranes (Pérez et al., 2005). Nevertheless, moderate resistance may occur (Boyer et al., 2007; Paris et al., 2010), underlying the possibility that, in some instances, *B. thuringiensis* var. *israelensis* might persist in the environment for a long time, enhancing the likelihood of evolving resistant mosquito populations. Indeed, *B. thuringiensis* var. *israelensis* has low survivability in the environment but, in specific conditions, it persists and proliferates (Tilquin et al., 2008; Melo-Santos et al., 2010).

*Lysinibacillus sphaericus* is a second, potent mosquitocidal bioinsecticide. During sporulation it produces two separate proteins, BinA (42 kDa) and BinB (51 kDa), that form a binary toxin which accumulates as parasporal crystalline inclusions (Alexander and Priest, 1990). Some strains also produce non-crystal mosquitocidal toxins (Mtx1, Mtx2, and Mtx3) during vegetative growth. Upon binding to brush border membranes of midgut cells, *L. sphaericus* toxins are internalized and induce cell death either via ADP-ribosylation, as is the case of Mtx1 toxin (Thanabalu et al., 1993), or activation of apoptosis (Tangsongcharoen et al., 2015). As binding of Bin toxin to midgut cells is mediated by a single receptor (maltase), resistance is easily acquired as a consequence of mutations in this toxin binding protein (Darboux et al., 2002). The insurgence of *L. sphaericus* resistant populations has been described since 1997 (Nielsen-Leroux et al., 1997) and resistance in field populations of various countries has been reported (Su et al., 2018, 2019).

The highly effective results obtained with *B. thuringiensis* var. *israelensis* and *L. sphaericus* led to several screening campaigns allowing the identification of other useful bacterial biopesticide species, such as *Chromobacterium subtsugae* (Martin et al., 2007), *Yersinia entomophaga* (Hurst et al., 2011), and *Brevibacillus laterosporus* (de Oliveira et al., 2004; Ruiu, 2013).

Many strains of *B. laterosporus* have been isolated and the list of insects susceptible to their entomopathogenic activity includes Coleoptera, Lepidoptera (de Oliveira et al., 2004), mosquitoes, black flies (Favret and Yousten, 1985; Rivers et al., 1991), and house flies (Ruiu et al., 2007). Its pathogenicity against Diptera has been associated to the characteristic canoe-shaped parasporal body (CSPB) which consists of four major proteins: CpbA, CpbB, CHRD, and ExsC (Marche et al., 2017). Ingestion of lysates of recombinant *Escherichia coli* strains expressing these proteins results in the death of house flies, implying their role as insecticidal toxins (Marche et al., 2017).

Here we report the isolation of a novel *B. laterosporus* strain with high toxicity against mosquito larvae. This strain was isolated in a screening campaign of bacterial isolates active against *Aedes albopictus*, a globally distributed invasive species vector of many mosquito-borne diseases (Paupy et al., 2009). We carried out morphological, genomic, and insecticidal characterization of the new isolate, and determined that its larvicidal activity is significantly higher than that of the LMG 15441 reference strain. Thus, the new isolate characterized in this paper can be considered as a candidate for the development of novel biocontrol formulations, alone or in combination with *B. thuringiensis* var. *israelensis* and *L. sphaericus* preparations to enhance their efficacy and avert the insurgence of resistance.

## Materials and Methods

### Bacterial Strains and Growth Media

*Lysinibacillus sphaericus* strain 1593 (*Bacillus* Genetic Stock Center ID 13A1; Priest et al., 1997), *B. laterosporus* strains LMG 15441 (*Bacillus* Genetic Stock Center ID 40A1; Rivers et al., 1991) and DSM25 (Shida et al., 1996) were used as positive controls. Strains 1593 and LMG 15441 were purchased from the *Bacillus* Genetic Stock Center (Columbus, OH, United States); strain DSM25 was purchased from DSMZ – German Collection of Microorganisms and Cell Cultures GmbH (Braunschweig, Germany). Growth media employed in this work include Luria–Bertani medium (LB), T3 medium (3 g/L tryptone, 2 g/L tryptose, 1.5 g/L yeast extract, 0.05 M sodium phosphate (pH 6.8), 0.005 g/L MnCl_2_) (Martin and Travers, 1989) and BP medium (7 g/L Bactopeptone, 6.8 g/L KH_2_PO_4_, 0.12 g/L MgSO_4_⋅7H_2_O, pH 7.4; BP medium is completed by addition of 10 μM MnSO_4_⋅4H_2_O, 50 μM ZnSO_4_⋅7H_2_O, 50 μM FeSO_4_, 100 μM CaCl_2_⋅4H_2_O, 0.3% glucose just before use] (Lecadet et al., 1980).

### Samples Collection, Screening, and Isolation of Bacteria With Larvicidal Activity Toward *Ae. albopictus*

Fourty-four soil samples were collected from different geographical areas [Supplementary Table 1: Italy (23), Cameroon (4), Zimbabwe (8), Philippines (3), United Kingdom (1), Cuba (1), Myanmar (1), Kenya (1), Pakistan (1), and Tajikistan (1)] for the isolation of bacteria with insecticidal activity toward larvae of *Ae. albopictus*. Soil samples (about 100 g each) were collected from 2 to 5 cm below the surface using a sterile spatula and transferred in sterile 50 mL Falcon tubes. Collected samples were transported to the laboratory and stored at 4°C.

A protocol for selecting and growing the sporulating and cultivable bacteria of each soil samples was developed. In order to enrich in entomopathogenic species, methods previously described for the isolation of *B. thuringiensis* from soil were combined and adapted (Travers et al., 1987; Santana et al., 2008; Patel et al., 2013). One gram of each sample was incubated at 80°C for 5 h before being inoculated in 20 mL LB medium supplemented with 0.25 M sodium-acetate. After 4 h of incubation at 30°C (200 rpm), 1 mL of culture was centrifuged at 1,000 rpm for 1 min to settle down soil particles. The supernatant was transferred into a sterile test tube and treated at 80°C for 10 min. Serial dilutions of each sample were spread on T3-agar and 0.25 mL of supernatant were used to inoculate 25 mL of T3 medium which is optimal for *B. thuringiensis* sporulation. After 48 h of growth at 30°C (200 rpm), each culture was diluted 50-fold in 25 mL of BP medium and incubated at 30°C, 200 rpm, for 72 h. BP is a complete medium for *B. thuringiensis* growth and sporulation. Appropriate dilutions (10^–4^, 10^–6^, 10^–7^, and 10^–8^) of each culture were spread onto T3-agar medium plates and the rest of the cultures were collected by centrifugation at 10,000 rpm for 10 min at 4°C. Pellets were washed three times with NaCl 1 M, EDTA 10 mM and twice with dH_2_O before being stored at −80°C or immediately used in larvicidal assays.

Cultures showing larvicidal activity were selected for further experiments. Colonies grown on T3-agar plates on which dilutions of active cultures had been spread were isolated to single colonies. Single clones were grown in 20 mL of BP for 72 h (30°C, 200 rpm) and pellets were collected, washed, and stored as described above before being assayed for their larvicidal activity.

### Larvicidal Assays and Determination of LC_50_

All experiments were performed using *Ae. albopictus* Rimini strain which was reared at 28°C, in a 12 h-light/12 h-dark photoperiod, with 70% of humidity.

The mixtures of cells and spores obtained after culture of soil inocula were screened for their larvicidal activity using 10 second-instar larvae of *Ae. albopictus* Rimini strain placed in 10 mL of dH_2_O without any nutritional supplement. Pellets were resuspended in dH_2_O and added at the final concentration of 2 g/L (expressed as biomass wet weight/litre). The same conditions were used in bioassays performed to screen individual bacteria isolates for potential larvicidal activity. In these screening assays, the concentration of 2 g/L was chosen to be able to detect activity even in soil samples with a low concentration of larvicidal bacteria and in bacterial isolates with low activity. A negative control group only exposed to distilled water was included in each experiment. Suspensions of *L. sphaericus* strain 1593 and *B. laterosporus* strain LMG 15441 collected after 72 h of growth in BP medium were used as positive controls. Mortality was recorded at 24 h intervals until 72 h from the beginning of the assay. The experiments were carried out at room temperature (22–25°C) in a laboratory location exposed to natural sunlight. Each test was performed in duplicate. Survival curves of each isolate were compared to those of the positive controls using a Long-Rank (Mantel–Cox) test in Prism GraphPad; *P*-values were corrected using False Discovery Rate.

Dose-response curves for strains SAM19, LMG 15441, and DSM25 were performed using 25 second-instar larvae of *Ae. albopictus* Rimini strain placed in 100 mL of dH_2_O. Larvae were exposed to serial dilutions (range from 320 to 5 mg/L) of cells-spores suspensions in distilled water, without adding food. For each strain, five to seven concentrations were tested alongside a negative control group. Each test was performed in duplicate (technical replicate) and the entire experiment was repeated three times. The number of cfu and spores per mL used in the assay was determined by plating appropriate dilutions of each suspension before and after heat treatment at 80°C for 10 min. The experiments were carried out at room temperature and mortality was recorded at 24 and 48 h. Mortality was calculated according to the following formula:

$$mortality\left(\%\right)=\frac{X-Y}{X}100$$

where *X* = percentage survival in the untreated control and *Y* = percentage survival in the treated sample.

Based on the obtained data, we performed a probit analysis using R studio (library ecotox and SciViews) and the results were visualized using ggplot2 (R Core Team, 2020).

### Identification of Isolates by PCR and 16S rRNA Sequencing

Selected bacterial isolates showing larvicidal activity against *Ae. albopictus* were initially identified by performing colony PCRs targeting conserved regions of genes encoding known entomopathogenic toxins of *L. sphaericus*, *B. thuringiensis* var. *israelensis* and *B. laterosporus*. Primers employed in this work are reported in Table 1. Genes encoding the mosquito larvicidal toxins BinA, BinB, Mtx1, Mtx2, and Mtx3 of *L. sphaericus* were amplified by colony multiplex PCR (Jagtap et al., 2009). The same approach was used for the detection of *B. thuringiensis* var. *israelensis* toxin genes *cry4*, *cry10*, and *cry11* (Vidal-Quist et al., 2009). For the identification of *B. laterosporus* strains, a set of primers targeting the gene encoding the conserved 28 kDa spore surface protein was employed (Marche et al., 2019). The taxonomic classification of SAM19 was determined by sequencing the V3–V4 hypervariable regions of the 16S rRNA gene. The fragment of interest was amplified using primers 16Sf (5′-CCTACGGGNGGCWGCAG) and 16Sr (5′-GACTACHVGGGTATCTAATCC) (Klindworth et al., 2013).

**TABLE 1:** Primers used for the detection of mosquitocidal toxin genes in the isolates with larvicidal activity against *Ae. albopictus*.

**Organism**	**Target gene(s)**	**Primer**	**Sequence (5′–3′)**	**Positive isolates**	**References**
*Bacillus thuringiensis* var *israelensis*	*cry4*	cry4f cry4r	GCATATGATGTAGCGAAACAAGCC GCGTGACATACCCATTTCCAGGTCC	–	Vidal-Quist et al., 2009
	*cry10*	cry10f cry10r	TATTGTTGGAGTTAGTGCAGGTATTATTGTAG TATTCCATGTTGCGTTAGTATTAGTTC	–	Alberola et al., 1999
	*cry11*	cry11f cry11r	TTAGAAGATACGCCAGATCAAGC CATTTGTACTTGAAGTTGTAATCCC	–	Vidal-Quist et al., 2009
*Lysinibacillus sphaericus*	*binA*	binAf binAr	CCAGAAAACGAGCAATACCC GACCACATGCTTTGCCAATA	–	Jagtap et al., 2009
	*binB*	binBf binBr	CCCCAAACATCCTTACTTGAGA GCGCACTTCCTTTAACTGCT	–	
	*mtx1*	mtx1f mtx1r	ATTCCCTCTTTTGCTTCTCCA AGCACTATGAGGTGTCCAAGG	CB35, CB50, TJ9	
	*mtx2*	mtx2f mtx2r	TGATTGCAAGTTTTTTGTTTG CAGATGCTTCCCCAGATGTTA	–	
	*mtx3*	mtx3f mtx3r	TAGCTTTCCAGATGCAGCAA CGAAGTCTCATTTGCCTGACT	–	
*Brevibacillus laterosporus*	*cpbA*	cpbAf	CTGCTACTAGTT GATCTAAG	CR1	Marche et al., 2019
		cpbAr	CTGATTGGTAGCT TAGGTA	CR4	
				LC1	
				LC2	
				NA2	
				NA6	
				SAM1	
				SAM2	
				SAM3	
				SAM14	
				SAM15	
				SAM19	

### Transmission Electron Microscopy

Samples were collected at different time points during growth in BP medium at 30°C and pellets were prefixed in Karnowsky’s fixative in cacodylate buffer (pH 7.2). After post-fixation in 2% OsO_4_ in 0.1 M cacodylate buffer for 1.5 h at 4°C, samples were washed, dehydrated through a progressive ethanol gradient, transferred to propylene oxide and embedded in Epon 812. Thin sections (80 nm) were stained with saturated uranyl acetate, followed by Reynolds lead citrate and examined with Zeiss EM900 transmission electron microscope at 80 kV.

### Genomic Analysis of SAM19

Genomic DNA was extracted from SAM19 using the DNeasy Blood and Tissue Kit (QIAGEN). DNA sequencing was performed by Illumina MiSeq with a Nextera-XT pair-end library. 1,308,373 read pairs were produced. After a preliminary quality check with FastQC (Wingett and Andrews, 2018), reads were assembled with SPAdes 3.10 (Bankevich et al., 2012). The genome was annotated with Prokka (Seemann, 2014). Accordingly, the genome assembly was polished by removing short (<500 bp) contigs bearing no annotated gene. The draft genome sequences of *B. laterosporus* SAM19 has been deposited in GenBank under accession number JADGMT000000000.

For phylogenomic analysis, the predicted protein sequences of a representative set of 12 *B. laterosporus* genomes, plus *Bacillus agri* as outgroup, were downloaded from NCBI. Maximum likelihood phylogenomic analyses were performed on a concatenated set of single copy orthogroups, as previously described (Floriano et al., 2018).

In order to perform comparative analyses on genome structure and content, the genome sequences of two closely related strains from phylogenomics (DSM25 and BGSP7) were downloaded from NCBI. After reciprocal reorientation of contigs in case of draft assemblies, synteny analyses were performed with MUMmer (Delcher et al., 2002), using the NUCmer aligner and filtering out alignments <1,000 bp and <80% identity. Average nucleotide identity (ANI) values were calculated with the Enveomics online suite (Rodriguez-R and Konstantinidis, 2016). Annotation of possible antimicrobial molecules was performed using the on-line software AntiSMASH (Antibiotics and Secondary Metabolite Analysis Shell; Medema et al., 2011) and BAGEL4 (de Jong et al., 2006).

Clusters of Orthology Groups (COGs) in the predicted proteins of SAM19 and the two references were predicted with the NCBI pipeline (Galperin et al., 2015), and the respective COG repertoires directly compared.

## Results

### Isolation of Bacterial Strains With Larvicidal Activity Against *Ae. albopictus*

**FIGURE 1:**
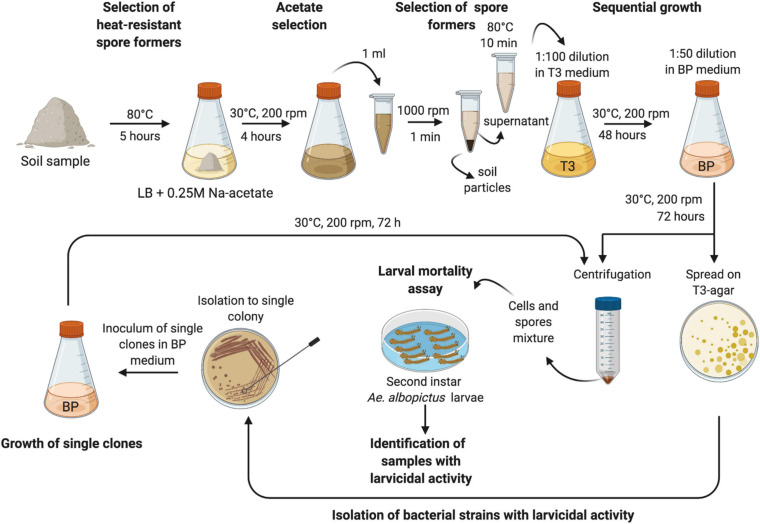
Schematic representation of the protocol used for the identification of soil samples enriched with bacteria with insecticidal activity and for the isolation of bacterial strains with larvicidal activity against *Ae. albopictus*. Created with BioRender.com.

**FIGURE 2:**
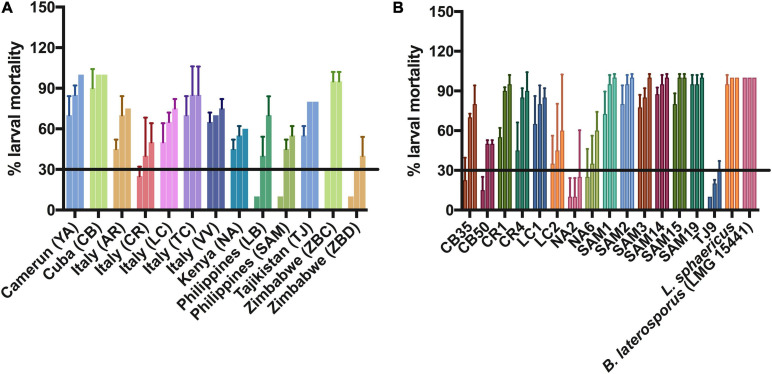
Percentage of *Ae. albopictus* larval mortality at 24, 48, and 72 h after treatment with mixtures of cells and spores of cultures originating from the inoculum of 13 soil samples **(A)** or of single bacterial isolates **(B)**. Suspensions were added at the final concentration of 2 g/L. For each sample, the first, second and third bar represent larval mortality at 24, 48, and 72 h, respectively. Under the employed conditions, treatment with *B. laterosporus* strain LMG 15441 induced 100% of larval mortality in all experiments [Standard deviation (SD) = 0]. Data are the average ± SD of two independent experiments, each performed in duplicate.

The sporulating and cultivable bacterial communities of 44 soil samples were grown and screened for their larvicidal activity against second instar larvae of *Ae. albopictus*. To this purpose, based on methods previously described for the isolation of *B. thuringiensis* from soil samples (Travers et al., 1987; Santana et al., 2008; Patel et al., 2013), a new strategy for the selection and cultivation of sporulating bacteria enriched in entomopathogenic strains was developed (described in section “Materials and Methods” and Figure 1). The mixtures of cells and spores obtained from each soil sample were resuspended in dH_2_O and used in larvicidal assays at the final concentration of 2 g/L (Figure 1). After 24 h of treatment, eight mixtures of soil bacteria (YA, CB, AR, LC, TC, VV, NA, and TJ) caused more than 30% larval mortality. Larvicidal activity increased over time and, 48 h after the beginning of the assay, five additional suspensions of soil bacteria (CR, LB, SAM, ZBC, and ZBD) killed at least 30% of *Ae. albopictus* larvae (Figure 2A). For each of the final 13 active samples, purified clones were grown in BP medium and the mixtures of cells and spores were harvested after 72 h of growth. Pellets were used in larvicidal assays at the final concentration of 2 g/L. After 24 h from the beginning of the assay, 10 bacterial clones isolated from three different soil samples induced more than 30% mortality against second instar larvae of *Ae. albopictus*. Five additional clones isolated from Cuba, Kenya, and Tajikistan also displayed larvicidal activity, but the percentage of larval mortality induced at 24 h was below 30% (Figure 2B). Larval mortality induced by each strain increased over time, reaching 100% in the case of clones isolated from soil collected in the Sampaloc lake area (Philippines) (Figure 2B). Furthermore, statistical analysis of the survival curves indicated that the Philippine isolates, with the exception of SAM3, were the only strains not being significantly different (*p* > 0.05) from the two positive controls (*L. sphaericus* and *B. laterosporus*), suggesting a similar larvicidal activity.

### Identification of the Mosquitocidal Isolates

In order to assess whether the isolated clones belonged to known entomopathogenic bacterial species, their genomic DNA was used as a template in PCR reactions targeting known entomopathogenic toxin-encoding genes of the species *B. thuringiensis* var. *israelensis*, *L. sphaericus*, and *B. laterosporus* (Table 1). While no amplification products were obtained using primers pairs annealing to conserved regions of the *B. thuringiensis* var. *israelensis* toxin genes *cry4*, *cry10*, and *cry11*, an amplicon corresponding to the *L. sphaericus mtx1* toxin gene was obtained in three (CB35, CB50, and TJ9) of the five clones with lower larvicidal activity (Figure 2B and Table 1). More interestingly, the use of primers targeting a gene encoding a highly conserved 28 kDa spore surface protein of *B. laterosporus* yielded an amplification product in all other active clones (Table 1; Marche et al., 2019).

Due to their low level of activity, the *L. sphaericus* isolates CB35, CB50, and TJ9 (Figure 2B) were not studied any further. Among the *B. laterosporus* clones, those isolated from soil collected in the Sampaloc lake area (Philippines, SAM) induced higher mortality; clone SAM19, displaying the highest larvicidal activity after 24 h of treatment, was selected for further characterization.

**FIGURE 3:**
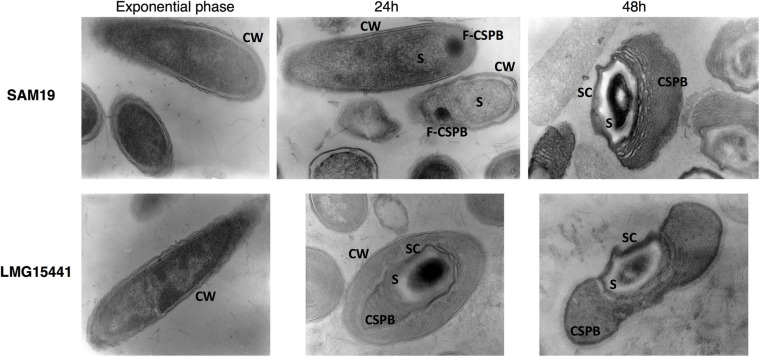
Transmission electron microscopy (TEM) images of *Brevibacillus laterosporus* strains SAM19 and LMG 15441 at different growth stages: Vegetative cell during exponential phase; Sporangium including forming spore at 24 h after inoculum; Free spore. CW, cell wall; S, spore; SC, spore coat; F-CSPB, forming canoe-shaped parasporal body; CSPB, canoe-shaped parasporal body.

BLAST analysis of its 16S rRNA gene sequence confirmed its classification as *B. laterosporus.* Cell and spore morphology were examined by transmission electron microscopy (TEM). To this purpose, SAM19 and the wild type *B. laterosporus* reference strain LMG 15441 were grown in synchronized cultures in BP medium and cultures were collected at different time points. As shown in Figure 3, during late stationary phase (24 h), an electrodense structure, probably related to the nascent parasporal body, could be observed at one pole of SAM19 spores, which were still contained within their mother cells. At 48 h, when sporulation was complete, a lamellar CSPB, firmly anchored to one side of the spore coat, was observed. The morphology of the mature spore appears similar to that of the reference strain LMG 15441 and to other *B. laterosporus* strains with entomopathogenic activity against Diptera (Marche et al., 2017).

### Genome Sequence and Phylogenomic Analysis of *B. laterosporus* SAM19

The final draft genome assembly suggests that the chromosome of SAM19 is 5,550,463 bp-long (174 contigs; N50 = 90,219 bp; L50 = 20, largest contig = 316,450 bp; GC = 40.1%): 5,252 protein coding genes and 108 ncRNA genes were annotated.

**FIGURE 4:**
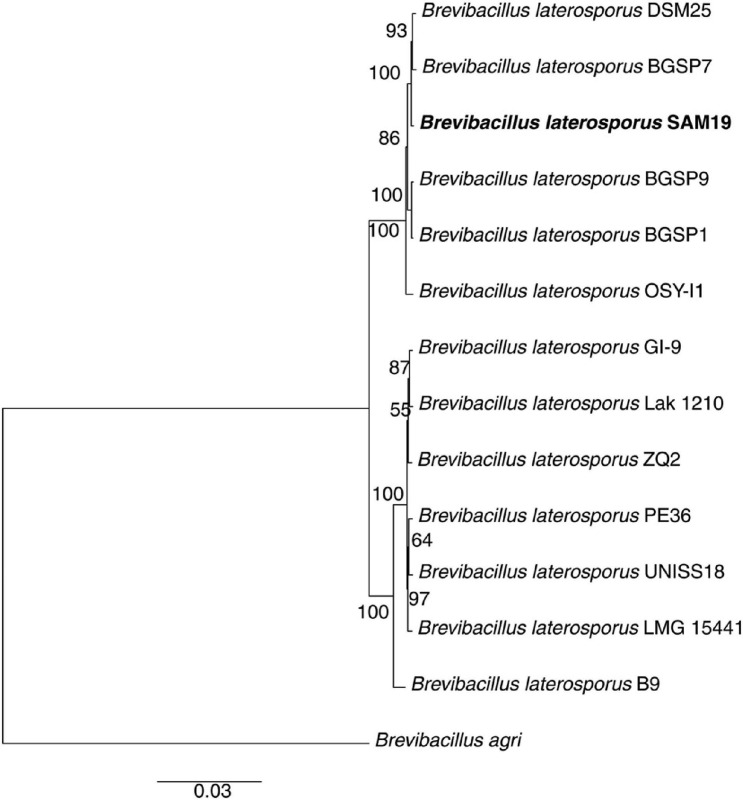
Maximum likelihood phylogenomic tree obtained with RAxML (Stamatakis, 2015) showing the relationships of SAM19 with representative *B. laterosporus* strains. Numbers of branches stand for bootstrap supports with 100 pseudo-replicates, scale bar for estimated proportional sequence divergence.

Phylogenomic analyses confirmed the assignment of SAM19 to the *B. laterosporus* species (Figure 4). In particular, this new isolate forms a monophyletic lineage (100% support) with strains BGSP7 and DSM25, being more closely related with BGSP7 (93% support). These two strains were thus selected for more detailed comparative analyses.

### Larvicidal Activity of *B. laterosporus* SAM19

**FIGURE 5:**
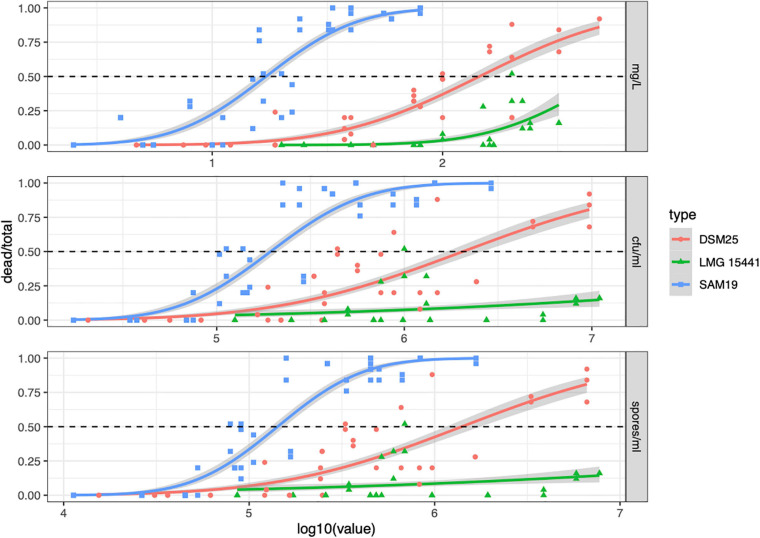
Probit analysis of mortality data of *Aedes albopictus* larvae treated with *Brevibacillus laterosporus* strains SAM19, DSM25, and LMG 15441. The analysis was performed on data collected at 24 h after exposure to bacteria. The dead/total ratio is reported as a function of the concentration (log10) of each suspension expressed as mg/L **(upper panel)**, cfu/mL **(middle panel)** or spores/mL **(lower panel)**.

The larvicidal activity of SAM19 was determined and compared with that of the phylogenetically proximal strain DSM25. Strain LMG 15441 was included in the analysis: despite being on a different phylogenetic branch with respect to SAM19, it shows phylogenetic proximity with UNISS18, a *B. laterosporus* strain with entomopathogenic activity (Ruiu et al., 2007; Figure 4). As reported in Figure 5, the lethal effects were concentration dependent and at 24 h post infection SAM19 displayed the highest larvicidal activity, with a LC_50_ of 10^1.233^ mg/L (confidential limits 10^1.154^–10^1.307^), corresponding to 10^5.287^ cfu/mL (10^5.192^–10^5.381^) and 10^5.153^ spores/mL (10^5.077^–10^5.229^) (Supplementary Table 2). SAM19 LC_50_ values did not change significantly after 48 h (Supplementary Figure 1 and Supplementary Table 2), confirming that the effect on larval viability is exerted within 24 h since exposure to the strain suspension. Interestingly, for all three strains LC_50_ values expressed as cfu/mL and spores/mL are similar to each other (Supplementary Table 2), consistent with the fact that larvicidal activity is associated with the spore.

### Gene Content Analysis

In order to investigate whether the lower LC_50_ of SAM19 could be associated to unique functions encoded by the new isolate, its genome was compared with those of DSM25 and BGSP7. Coherently with phylogenetic proximity, SAM19 showed a very high level of synteny (14 and 8 inversions) and an ANI of 99.11 and 99.13% with DSM 25 and BGSP7, respectively (Supplementary Figure 2).

93.3% of the 1,910 COGs present on the genome of SAM19 were shared with the other two strains; only 75 were unique to this new isolate. BLAST analysis of the corresponding proteins revealed that homologues with >95% sequence identity were present in other *B. laterosporus* strains. No clear candidates with a potential involvement in virulence could be identified (Supplementary Datasheet 1).

As SAM19 is phylogenetically close to the antimicrobials producer strain BGSP7, its genome was also searched for the presence of genes coding for secondary-metabolites biosynthesis and bacteriocins using the online bioinformatic tools AntiSMASH (Antibiotics and Secondary Metabolite Analysis Shell; Medema et al., 2011) and BAGEL4 (de Jong et al., 2006). Non-ribosomal peptide synthetase clusters encoding bogorol A and brevicidine were identified by AntiSMASH analysis. Moreover, SAM19 genome displayed the potential to produce numerous bacteriocins, including laterosporulin, sactipeptides, UviB, and lanthipeptide class I (Supplementary Table 3).

## Discussion

*Bacillus thuringensis* is the most widely employed biopesticide. Its high insecticidal activity, the specificity of each subspecies for a limited clade of insects (Ehling-Schulz et al., 2019), its low environmental impact and the possibility of using genes encoding toxic proteins to generate transgenic organisms make the use of *B. thuringiensis* a successful strategy to control insect pests. Currently, there are more than 98 formulated bacterial pesticides commercially available (Lacey et al., 2015).

The two main pitfalls of the large use of *B. thuringiensis* are its short lifetime and concerns about public health. As the stability of insecticidal crystalline proteins is hampered by solar radiation, effective control of insect pests requires continuous spraying of *B. thuringensis* formulations. Furthermore, *B. thuringensis* belongs to the *Bacillus cereus* group together with *Bacillus cereus sensu stricto*, *Bacillus anthracis, Bacillus weihenstephanensis, Bacillus mycoides, Bacillus pseudomycoides, Bacillus cytotoxicus*, and *Bacillus toyonensis*. *B. cereus* is recognized to secrete the emetic toxin cereulide, coded by the *ces* operon (Agata et al., 1995). Although there is no evidence that *B. thuringiensis* secretes it, concerns were raised by the European Food Safety Authority on the possibility that some *B. thuringensis* strains might contain genes for its synthesis, or that some formulations might contain other *B. cereus* species, requiring the full sequencing of the *B. thuringensis* strains used (EFSA Panel on Biological Hazards (Biohaz), 2016). Notably, *ces* genes are located in a transposable element in *B. weihenstephanensis* (Mei et al., 2014), raising questions about the possible horizontal transfer between species.

In view of these premises, the search for possible alternative biopesticides is increasing, particularly to control vector-borne diseases. It is worth noting that vector control is still the most effective strategy for the prevention of many insect-borne diseases, particularly for mosquito-borne diseases, including malaria, dengue and Zika. Their endemic area are the tropical and subtropical regions, but climate change and intense human commercial activities are expanding the geographical distribution of many mosquito species, allowing the transmission of mosquito-borne diseases in new countries. This is the case of *Ae. albopictus*, which spread from South-East Asia to all over the world and which caused in the last decade several outbreaks of chikungunya and dengue in Europe (Benedict et al., 2007; Gossner et al., 2018).

In this context, this work was aimed at isolating novel cultivable bacterial strains with insecticidal activity toward larvae of *Ae. albopictus*. To this purpose, a novel method to rapidly identify soil samples colonized by bacteria with larvicidal activity was developed. In order to have a collection as diversified as possible, samples were collected from different areas of Italy, from African (Kenya, Zimbabwe, and Camerun) and Asian countries (Pakistan, Tajikistan, Philippines, and Myanmar) as well as from Cuba and the United Kingdom. Different environments such as city, rural areas, agricultural lands, river basins, and lake areas were sampled (Supplementary Table 1). The classical protocol for the isolation of strains of *B. thuringiensis* var. *israelensis* is based on acetate selection followed by heat treatment: soil is inoculated in rich liquid medium supplemented with 0.25 M sodium acetate and, after 4 h of incubation at 30°C with aeration, culture supernatant is heated at 80°C. Since *B. thuringiensis* spores do not germinate in the presence of 0.25 M sodium acetate, germinated bacteria of species other than *B. thuringiensis* are heat killed (Travers et al., 1987). However, since *B. thuringiensis* concentration in soil can be very low, this strategy was reported to have low efficiency and alternative methodologies, including dry-heat pre-treatment of soil samples and enrichment method, were developed (Santana et al., 2008; Patel et al., 2013). All these methods require microscopic examinations of single clones for their ability to produce spores and crystals after at least 72 h of growth on sporulation agar-medium. Isolating, cultivating, and assaying the larvicidal activity of a high number of single clones for each soil sample is a time-consuming procedure. To overcome this limitation, we developed a new two steps procedure. In the first step, a mixed population of sporulating cultivable bacteria was obtained and tested for its larvicidal capacity. In the second step, single bacterial clones obtained from active soil samples were tested for their entomopathogenic activity. This method provides two major advantages: (i) multiple soil samples can be assayed in parallel and (ii) the bacterial population obtained from each soil contains a high percentage of active bacteria. We cannot exclude that entomopathogenic bacteria could be present at low density even in soil samples that displayed low or null larvicidal activity under employed conditions. Surprisingly, the majority of active clones were not *B. thuringiensis* var. *israelensis* strains but were identified as *L. sphaericus* and *B. laterosporus*, two known entomopathogenic species. While *L. sphaericus* isolates showed lower activities compared to the control reference strain 1593, isolated *B. laterosporus* strains displayed high toxicity.

*Brevibacillus laterosporus* has attracted increasing attention as a producer of antimicrobial compounds and secondary metabolites and is used as probiotic for humans (Ruiu, 2013). Importantly, strains with insecticidal activities against Diptera, Lepidoptera, and Coleoptera have been reported, making it an important candidate for the biocontrol of different pests with very limited risks for public health (de Oliveira et al., 2004; Ruiu, 2013).

The genome of the newly isolated strain was sequenced and the phylogenomic analysis revealed its close proximity to strains DSM25 and BGSP7. DSM25 is a known entomopathogen active against *Culex quinquefasciatus* and *Aedes aegypti* (Favret and Yousten, 1985; Rivers et al., 1991). BGSP7 was recently isolated from silage and selected for its ability to produce antimicrobial molecules active against Gram-negative (*Klebsiella pneumoniae* Ni9 and *Pseudomonas aeruginosa* MMA83) and Gram-positive (*Staphylococcus aureus* ATCC25923 and *Listeria monocytogenes* ATCC19111) multi-drug resistant pathogens (Miljkovic et al., 2019). Moreover, antifungal activity and toxicity toward larvae and adults of the potato beetle *Leptinotarsa decemlineata* were reported for BGSP7 (Miljkovic et al., 2019).

Even though the possible antimicrobial activity of SAM19 was not investigated in this work, bioinformatic analysis of its genome provided evidence of the presence of genes encoding antimicrobials.

The genome of SAM19 revealed the presence of 75 COGs not shared with its closest relatives DSM25 and BGSP7. However, analysis of the corresponding proteins did not highlight any specific feature suggesting higher toxicity of SAM19 compared to DSM25. Interestingly, SAM19 unique COGs included different phage related proteins, suggesting the possibility that phage functions and lysogenic conversion could play a role in the increased entomopathogenic activity.

The insecticidal activity of some strains of *B. laterosporus* has been associated to the presence of cytoplasmic inclusions containing insecticidal crystal proteins (Orlova et al., 1998). As revealed by TEM observations, SAM19 does not display intracellular crystalline inclusions. Entomopathogenic *B. laterosporus* strains lacking parasporal crystals have been previously described. Among these, the strain UNISS18 is characterized by insecticidal activity against *Musca domestica* and *Ae. aegypti* (Ruiu et al., 2007). This strain, however, is phylogenomically distant from SAM19. Its pathogenicity has been correlated to different virulence factors, including two surface proteins, CbpA and CbpB, associated to the CSPB. While a gene (LICBGMMG_04116) encoding a protein with 91.89% identity to UNISS18 CpbA was identified in SAM19, the new isolate did not display any CpbB homologue. A BLAST search analysis revealed that SAM19 encodes proteins previously identified as putative virulence factors expressed by UNISS18 during growth in the insect body (Marche et al., 2018). These proteins, with a high level of sequence identity (81.41–100%) with their UNISS18 homologues (Supplementary Table 4) include chitinases ChiA and ChiB (LICBGMMG_02893 Chitinase A1; LICBGMMG_01497 Chitodextrinase), a collagenase-like protease PrtC (LICBGMMG_01889), GlcNAc-binding protein (LICBGMMG_01411), protective antigen proteins (LICBGMMG_05212; LICBGMMG_04772; LICBGMMG_05296), bacillolysin (LICBGMMG_04689), thermophilic serine proteinase (LICBGMMG_02557), and the insecticidal toxin Mtx (LICBGMMG_04980 Epsilon-toxin type B). The toxicity of the new isolate against *Ae. albopictus* could therefore result from the concerted activity of a variety of virulence factors.

Further studies are needed to better characterize the toxicity of the newly identified SAM19 *B. laterosporus* strain against other mosquito and, more generally, Diptera species and to test the strain stability in field settings, using formulated or unformulated preparations. The higher toxicity of SAM19, compared with other *B. laterosporus* strains used as reference, supports the possibility of adding this strain to the arsenal of eco-compatible tools that can be used to control mosquitoes and to limit the spread of invasive species and their associated diseases. Noteworthy, since *B. laterosporus* strains have been previously described for their toxicity to insects (Coleoptera, Lepidoptera, and Diptera), nematodes and mollusks (Ruiu, 2013), it will be important to assess possible off-target effects of SAM 19 to other species and organisms. Furthermore, the identification of genes encoding antimicrobial molecules suggests its potential use for antimicrobial production.

## Data Availability Statement

The datasets presented in this study can be found in online repositories. The names of the repository/repositories accession number(s) can be found below: https://www.ncbi.nlm.nih.gov/genbank/, JADGMT000000000; https://www.ncbi.nlm.nih.gov/biosample/, SAMN16515084.

## Author Contributions

GB, PG, EF, GG, and AA contributed to the conception and design of the study. GB, SM, PG, VS, and EU screened the collection of soil samples, isolated bacterial strains with larvicidal activity against *Ae. albopictus*, and identified and characterized SAM19. CF, MC, and DS performed SAM19 genome assembly, annotation, and phylogenomic analysis. GR, EC, and LS performed TEM analysis of SAM19. GB, PG, MC, and DS drafted the manuscript. GB, PG, and EF finalized the manuscript. All authors read and approved the final manuscript.

## Conflict of Interest

The authors declare that the research was conducted in the absence of any commercial or financial relationships that could be construed as a potential conflict of interest.
