# Supplementary material for: Identification of a Novel Brevibacillus laterosporus Strain With Insecticidal Activity Against Aedes albopictus Larvae
Source: Front Microbiol. 2021 Feb 17;12:624014. doi: 10.3389/fmicb.2021.624014 (PMC7925996; doi:10.3389/fmicb.2021.624014)
Supplement: Supplementary file 1 [file Table_1.docx]

**Supplementary Table 1.** Collection sites and sampling date of soil samples used in this study.

| **Sample name** | **Country** | **Collection site** | **GPS coordinates** | **Collection date** |
| --- | --- | --- | --- | --- |
| **AR** | Italy | Arco – city area | N 45°55'0.693'' E 10°52'25.854'' | May 2015 |
| **AR1** | Italy | Arco – city area | N 45°54'20.931'' E 10°51'56.322'' | May 2015 |
| **AR2** | Italy | Arco – city area | N 45°54'53.428'' E 10°53'11.857'' | May 2015 |
| **AR3** | Italy | Arco – city area | N 45°55'5.685'' E 10°53'8.003'' | May 2015 |
| **AR4** | Italy | Arco – city area | N 45°55'19.764'' E 10°53'24.495'' | May 2015 |
| **AR5** | Italy | Arco – city area | N 45°55'6.62'' E 10°53'5.471'' | May 2015 |
| **BC** | Italy | Buonconvento -rural area | N 43°9'20.713'' E 11°28'20.905'' | June 2015 |
| **CR** | Italy | Correggio – agricultural area | N 44°45'26.334'' E 10°45'37.255'' | October 2015 |
| **LC** | Italy | Pavia – river basin | N 45°10'53.14'' E 9°9'17.175'' | November 2015 |
| **LG** | Italy | Pavia -river basin | N 45°10'53.18'' E 9°9'17.117'' | November 2015 |
| **MZA** | Italy | Mezzolombardo – city area | N 46°12'48.498'' E 11°5'47.793'' | May 2015 |
| **MZB** | Italy | Mezzolombardo – city area | N 46°12'47.172'' E 11°5'35.501'' | May 2015 |
| **MZC** | Italy | Mezzolombardo – city area | N 46°12'29.697'' E 11°5'29.59'' | May 2015 |
| **SCC** | Italy | San Cataldo – rural area | N 37°28'47.012'' E 13°58'47.546'' | January 2016 |
| **SCL** | Italy | San Cataldo – agricultural area | N 37°30'48.28'' E 13°57'28.962'' | January 2016 |
| **TC** | Italy | Pavia – river basin | N 45°10'46.132'' E 9°9'32.905'' | November 2015 |
| **TRA** | Italy | Trento – city area | N 46°3'5.442'' E 11°7'1.582'' | May 2015 |
| **TRB** | Italy | Trento – city area | N 46°3'5.025'' E 11°7'54.769'' | May 2015 |
| **TRC** | Italy | Trento – city area | N 46°3'9.024'' E 11°7'34.833'' | May 2015 |
| **TRD** | Italy | Trento – city area | N 46°3'24.028'' E 11°8'15.201'' | May 2015 |
| **TRE** | Italy | Trento – city area | N 46°3'54.465'' E 11°7'53.178'' | May 2015 |
| **VV1** | Italy | Vigolo Vattaro - rural area | N 46°0'14.115'' E 11°11'23.26'' | June 2015 |
| **VV2** | Italy | Vigolo Vattaro – city area | N 46°0'15.947'' E 11°11'33.04'' | June 2015 |
| **BI** | Myanmar | Yangon – city area | N 16°49'10.43'' E 96°11'0.909'' | October 2015 |
| **CB** | Cuba | La Habana- rural area | N 23°10'47.4'' O 82°12'21.2'' | August 2015 |
| **LB** | Philippines | Los Baños – city area | N 14°9'57.083'' E 121°14'36.077'' | October 2015 |
| **LBU** | Philippines | Los Baños – city area | N 14°10'2.217'' E 121°14'36.171'' | October 2015 |
| **SAM** | Philippines | San Pablo - Sampaloc Lake | N 14°4'53.1'' E 121°20'2.4å'' | October 2015 |
| **LO** | UK | London – West Acton-city area | N 51°31'8.561'' O 0°17'3.846'' | September 2015 |
| **NA** | Kenya | Nairobi -city area | S 1°17'44.271'' E 36°49'8.241'' | October 2015 |
| **PK** | Pakistan | Islamabad - city area | N 33°41'47'' E 73°1'15.012'' | October 2015 |
| **TJ** | Tajikistan | Dushanbe - city area | N 38°34'29.396'' E 68°47'6.096'' | November 2015 |
| **YA** | Cameroon | Yaounde - city area | N 3°51'26.783'' E 11°30'3.013'' | September 2016 |
| **YA4** | Cameroon | Yaounde- city area | N 3°31'9.972'' E 11°30'41.597'' | September 2016 |
| **YA5** | Cameroon | Batoufam – rural area | N 5°16'36.71'' E 10°28'2.211'' | September 2016 |
| **YA6** | Cameroon | Yaounde - city area | N 3°50'31.508'' E 11°29'12.601'' | September 2016 |
| **ZBA** | Zimbabwe | Beitbridge – rural area | S 22°9'49.796'' E 29°31'10.32'' | October 2015 |
| **ZBB** | Zimbabwe | Mutoko – rural area | S 17°23'56'' E 32°13'0.999'' | October 2015 |
| **ZBC** | Zimbabwe | Beitbridge – rural area | S 22°5'17'' E 29°18'34'' | October 2015 |
| **ZBD** | Zimbabwe | Beitbridge – rural area | S 22°11'3.025'' E  29°22'15.919'' | October 2015 |
| **ZBE** | Zimbabwe | Hwange – rural area | S 18°57'49’’ E  26°57'9'' | October 2015 |
| **ZBF** | Zimbabwe | Beitbridge – rural area | S 22°7'18'' E  29°23'5'' | October 2015 |
| **ZBH** | Zimbabwe | Harare – city area | S 17°49'27.644'' E 31°2'46.693'' | October 2015 |
| **ZBI** | Zimbabwe | Harare – city area | S 17°48'56.686'' E 31°3'26.801'' | October 2015 |
