# Supplementary material for: Identification of a Novel Brevibacillus laterosporus Strain With Insecticidal Activity Against Aedes albopictus Larvae
Source: Front Microbiol. 2021 Feb 17;12:624014. doi: 10.3389/fmicb.2021.624014 (PMC7925996; doi:10.3389/fmicb.2021.624014)
Supplement: Supplementary file 2 [file Table_2.docx]

**Supplementary Table 2.** LC_50_ with upper and lower confidence limits of strains LMG 15441, SAM19 and DSM25 at 24h after the beginning of the assay. LC_50_ values are expressed as concentration of bacterial suspension (mg/L), as cfus/ml and as spores/ml. Note that LC_50_ of LMG 15441 is not included in the tested concentration range and it is therefore estimated based on the available data.

|  |  | **LC_50_** (LCL - UCL) | |
| --- | --- | --- | --- |
|  |  | **24 h** | **48 h** |
| **mg/L**  (log10) | LMG 15441 | **2.720** (2.537-3.307) | **2.716** (2.549-3.053) |
|  | SAM19 | **1.233** (1.154-1.307) | **1.143** (1.073-1.209) |
|  | DSM25 | **2.160** (2.067-2.280) | **1.969** (1.841-2.130) |
| **cfu/ml** (log10) | LMG 15441 | **9.787** (7.442-nd) | **7.925** (7.153-11.741) |
|  | SAM19 | **5.287** (5.192-5.381) | **5.167** (5.100-5.231) |
|  | DSM25 | **6.312** (6.140-6.554) | **6.076** (5.932-6.262) |
| **spores/ml** (log10) | LMG 15441 | **9.970** (7.327-nd) | **7.815** (7.006-11.941) |
|  | SAM19 | **5.153** (5.077-5.229) | **5.073** (5.000-5.145) |
|  | DSM25 | **6.146** (5.977-6.383) | **5.949** (5.795-6.153) |
