# Supplementary material for: Identification of a Novel Brevibacillus laterosporus Strain With Insecticidal Activity Against Aedes albopictus Larvae
Source: Front Microbiol. 2021 Feb 17;12:624014. doi: 10.3389/fmicb.2021.624014 (PMC7925996; doi:10.3389/fmicb.2021.624014)
Supplement: Supplementary file 3 [file Table_3.docx]

**Supplementary Table 3.** Genome-based identification of molecules with antimicrobial activity by bioinformatic tools (BAGEL3). For each protein, the percentage of aminoacid sequence identity with the corresponding BGSP7 homologue (Miljkovic *et al.*, 2019) is reported.

| **Antimicrobial molecule** | **SAM19 annotated gene** | **% aminoacid sequence identity with the corresponding homologue in strain BGSP7** |
| --- | --- | --- |
| Laterosporulin | LICBGMMG_05104 hypothetical protein | 73.08% |
| Linocin M18 | LICBGMMG_02258 Maritimacin | 100% |
| Sactipeptide | LICBGMMG_00122 hypothetical protein | 100% |
| UviB | LICBGMMG_00729 hypothetical protein | 100.00% |
|  | LICBGMMG_04741 hypothetical protein | 85.06% |
|  | LICBGMMG_05143 hypothetical protein | 86.21% |
|  | LICBGMMG_00259 hypothetical protein | 82.76% |
|  | LICBGMMG_02477 hypothetical protein | 64.37% |

Miljkovic, M., Jovanovic, S., O'Connor, P.M., Mirkovic, N., Jovcic, B., Filipic, B. et al. (2019). *Brevibacillus laterosporus* strains BGSP7, BGSP9 and BGSP11 isolated from silage produce broad spectrum multi-antimicrobials. PLoS One 14, e0216773. doi: 10.1371/journal.pone.0216773
