# Supplementary material for: Identification of a Novel Brevibacillus laterosporus Strain With Insecticidal Activity Against Aedes albopictus Larvae
Source: Front Microbiol. 2021 Feb 17;12:624014. doi: 10.3389/fmicb.2021.624014 (PMC7925996; doi:10.3389/fmicb.2021.624014)
Supplement: Supplementary file 4 [file Table_4.docx]

**Supplementary Table 4.** SAM19 homologues of putative virulence factors identified in *B. laterosporus* strain UNISS18 (Marche *et al.*, 2018)

| **Category** | **UNISS 18** | | **SAM19** | **Aminoacid sequence identity** |
| --- | --- | --- | --- | --- |
|  | **gene** | **protein** | **gene** |  |
| chitinase ChiA | MG725829.1 | AWK77732.1 | LICBGMMG_02893 Chitinase A1 | 92.14% |
| chitinase ChiD | MG725830.1 | AWK77733.1 | LICBGMMG_01497 Chitodextrinase | 90.32% |
| collagenase-like protease PrtC | MG725831.1 | AWK77734.1 | LICBGMMG_01889 putative protease YdcP | 98.79% |
| GlcNAc-binding protein (Gbp) | MG725832.1 | AWK77735.1 | LICBGMMG_01411 GlcNAc-binding protein A | 92.84% |
| protective antigen protein PA1 | MG725833.1 | AWK77736.1 | LICBGMMG_05212 Protective antigen | 87.82% |
|  |  |  | LICBGMMG_04772 Protective antigen | 83.89% |
|  |  |  | LICBGMMG_05296 Protective antigen | 81.68% |
| protective antigen protein PA2 | MG725834.1 | AWK77737.1 | LICBGMMG_05212 Protective antigen | 83.11% |
|  |  |  | LICBGMMG_04772 Protective antigen | 81.41% |
|  |  |  | LICBGMMG_05296 Protective antigen | 81.96% |
| bacillolysin (Bl18) | MG725835.1 | AWK77738.1 | LICBGMMG_04689 Bacillolysin | 94.33% |
| thermophilic serine proteinase (Tsp) | MG725836.1 | AWK77739.1 | LICBGMMG_02557 Thermophilic serine proteinase | 100% |
| spore surface proteins CpbA | KY124461.1 | AQX44450.1 | LICBGMMG_04116 hypothetical protein | 91.89% |
| spore surface proteins CpbB | KY124462.1 | AQX44451.1 | LICBGMMG_03902 hypothetical protein | 27.43% |
| insecticidal toxin Mtx | MG725837.1 | AWK77740.1 | LICBGMMG_04980 Epsilon-toxin type B | 96.53% |

Marche, M.G., Camiolo, S., Porceddu, A., Ruiu, L. (2018). Survey of *Brevibacillus laterosporus* insecticidal protein genes and virulence factors. J. Invertebr. Pathol. 155, 38-43. doi: 10.1016/j.jip.2018.05.002
