# Supplementary figures and images for: Identification of a Novel Brevibacillus laterosporus Strain With Insecticidal Activity Against Aedes albopictus Larvae
Source: Front Microbiol. 2021 Feb 17;12:624014. doi: 10.3389/fmicb.2021.624014 (PMC7925996; doi:10.3389/fmicb.2021.624014)

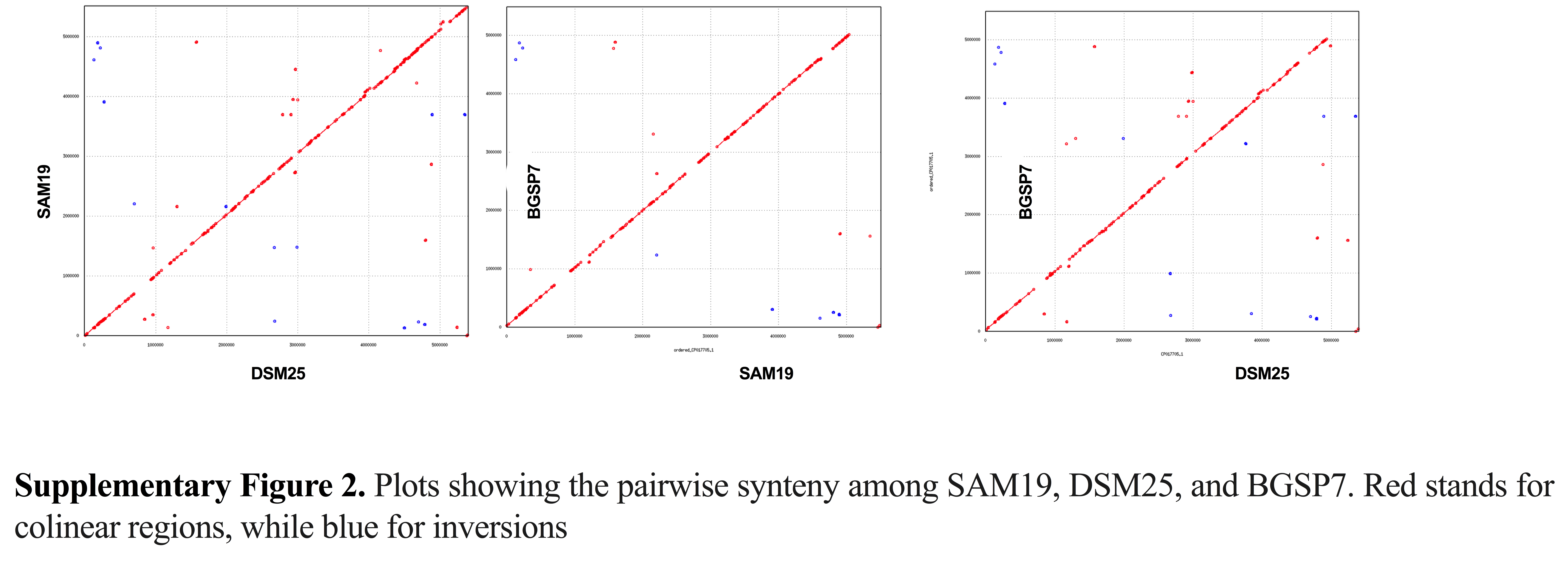

Supplement: Supplementary file 7 [file Image_2.tiff]
